# The Woodbridge Treatment of Typhoid Fever

**Published:** 1898-10

**Authors:** 


### The Woodbridge Treatment of Typhoid Fever.

DIRECTIONS.

At any stage of an attack of typhoid fever (the earlier the better), or in any pathological condition in which a general or intestinal antiseptic or eliminant is indicated, Dr. Woodbridge recommends that the treatment be begun with the following tablets, each containing:

| | |
|---|---|
| ℞ No. 1.—Podophyllum resin | 1-960 grain. |
| Mercurous chloride, mild | 1-16 grain. |
| Guaiacol carbonate | 1-16 grain. |
| Menthol | 1-16 grain. |
| Eucalyptol | q. s. |

One tablet of this formula should be given every fifteen minutes *during all the wakeful portion of the first forty-eight hours*, unless too frequent evacuations of the bowels demand their temporary discontinuance.

At the end of the twenty-four hours begin with formula No. 2, each tablet of which contains:

| | | |
|---|---|---|
| ℞ No. 2.— | Podophyllum resin | 1-960 grain. |
| | Mercurous chloride, mild | 1-16 grain. |
| | Guaiacol carbonate | 1-4 grain. |
| | Menthol | 1-16 grain. |
| | Thymol | 1-16 grain. |
| | Eucalpytol | q. s. |

One tablet of this formula should be given every fifteen minutes. This, formula, as well as formula No. 1, should be administered as freely as possible until not less than five or six free evacuations of the bowels have been secured during the second twenty-four hours of treatment or as soon as practicable thereafter, even though three or four or more of tablets No. 1 and No. 2 have been given every fifteen minutes.

As soon as these free evacuations of the bowels have been secured, the tablets of formula No. 1 should be discontinued and the intervals between the doses of tablets No. 2 so lengthened as to allow the movements of the bowels to become less and less frequent until reduced to one or two each day by the time the temperature has touched normal.

On the morning of the third or fourth day, soluble elastic capsules of formula No. 3 should be commenced, each capsule containing:

| | | |
|---|---|---|
| ℞ No. 3.— | Guaiacol carbonate | 3 grains. |
| | Thymol | 1 grain. |
| | Menthol | 1-2 grain. |
| | Eucalpytol | 5 minims. |

One capsule should be given every three or four hours, alternating with the tablets. On the fourth or fifth day the tablets should be discontinued for a day or two, and teaspoonful doses of a saturated solution of chlorate of potassium be administered every three hours during this period, after which tablets No. 2 should again be exhibited as freely as possible without causing too frequent evacuation of the bowels.

The patient should be carefully watched; and should the slightest symptoms of ptyalism supervene, the tablets should again be discontinued and the chlorate of potassa again freely exhibited.

### FOR CHILDREN.

In the treatment of typhoid fever in young children, tablets of formula No. 4 may be substituted for tablets of formula No. 1, each of the former containing:

℞ No. 4.—Podophyllum resin.....................1-960 grain.
Mercurous chloride, mild............ 1-16 grain.
Guaiacol carbinate...................... 1-8 grain.
Menthol.................................... 1-96 grain.
Eucalyptol... ........................... q. s.

One of these tablets should be given every hour or oftener to infants and young children, *pro re nata*—the object being to produce the same general effect as advised in the treatment of the disease in adults.

After which capsules of formula No. 5 should be administered, each of which contains:

℞ No. 5.—Guaiacol carbonate..................... 1-2 grain.
Thymol........................................ 1-8 grain.
Menthol.................................. 1-16 grain.
Eucalyptol................................ 1 minim.
Olive oil.................................... q. s.

Give one capsule three times a day or oftener, according to the age of the child.

During all the course of the treatment, in both children and adults, the patient must wash down each and every dose of medicine with large draughts of distilled water or, if indicated, some good laxative or diuretic mineral water.

If this treatment is begun early in the course of the disease, no other medicine will be needed, and if intelligently carried out will rarely, if ever, fail to abort an attack of typhoid fever.

The Woodbridge Treatment is put up in a case containing four bottles of 100 tablets each, No. 1; two bottles of 100 tablets each, No. 2; three boxes of one dozen each, soluble elastic capsules, No. 3.

The several formulæ are also supplied as follows: Tablets in bottles of 100 and 1000; capsules in boxes of one dozen and 100.

The above tabules are manufactured by Parke, Davis & Co., Detroit.

